# Palmitoylation-dependent control of JAK1 kinase signaling governs responses to neuropoietic cytokines and survival in DRG neurons

**DOI:** 10.1016/j.jbc.2023.104965

**Published:** 2023-06-24

**Authors:** Luiselys M. Hernandez, Audrey Montersino, Jingwen Niu, Shuchi Guo, Bulat Faezov, Shaun S. Sanders, Roland L. Dunbrack, Gareth M. Thomas

**Affiliations:** 1 Shriners Hospitals Pediatric Research Center (Center for Neurorehabilitation and Neural Repair), Lewis Katz School of Medicine at Temple University, Philadelphia, Pennsylvania, USA; 2 Institute for Cancer Research, Fox Chase Cancer Center, Philadelphia, Pennsylvania, USA; 3 Kazan Federal University, Kazan, Russian Federation; 4 Department of Neural Sciences, Lewis Katz School of Medicine at Temple University, Philadelphia, Pennsylvania, USA

**Keywords:** protein palmitoylation, Janus kinase (JAK), signal transduction, dorsal root ganglia, neuroscience

## Abstract

Janus Kinase-1 (JAK1) plays key roles during neurodevelopment and following neuronal injury, while activatory *JAK1* mutations are linked to leukemia. In mice, *Jak1* genetic deletion results in perinatal lethality, suggesting non-redundant roles and/or regulation of JAK1 for which other JAKs cannot compensate. Proteomic studies reveal that JAK1 is more likely palmitoylated compared to other JAKs, implicating palmitoylation as a possible JAK1-specific regulatory mechanism. However, the importance of palmitoylation for JAK1 signaling has not been addressed. Here, we report that JAK1 is palmitoylated in transfected HEK293T cells and endogenously in cultured Dorsal Root Ganglion (DRG) neurons. We further use comprehensive screening in transfected non-neuronal cells and shRNA-mediated knockdown in DRG neurons to identify the related enzymes ZDHHC3 and ZDHHC7 as dominant protein acyltransferases (PATs) for JAK1. Surprisingly, we found palmitoylation minimally affects JAK1 localization in neurons, but is critical for JAK1’s kinase activity in cells and even *in vitro*. We propose this requirement is likely because palmitoylation facilitates transphosphorylation of key sites in JAK1’s activation loop, a possibility consistent with structural models of JAK1. Importantly, we demonstrate a leukemia-associated *JAK1* mutation overrides the palmitoylation-dependence of JAK1 activity, potentially explaining why this mutation is oncogenic. Finally, we show that JAK1 palmitoylation is important for neuropoietic cytokine-dependent signaling and neuronal survival and that combined *Zdhhc3/7* loss phenocopies loss of palmitoyl-JAK1. These findings provide new insights into the control of JAK signaling in both physiological and pathological contexts.

Janus kinase-1 (JAK1) is the founding member of the JAK family of tyrosine kinases, which consists of JAK1-3 and the related Tyrosine kinase-2 (Tyk2) (1, 2). JAK1 appears to play critical roles that cannot be compensated for by other JAK family members, despite their broad expression. In particular, *Jak1* knockout (KO) mice die perinatally and multiple *Jak1* KO primary cell types fail to respond to an array of neuropoietic cytokines, such as Leukemia Inhibitory Factor (LIF) (3, 4). Among these cell types, dorsal root ganglion (DRG) sensory neurons appear to be particularly reliant upon JAK1 (4); DRG neuron numbers are greatly reduced in *Jak1* KO mice and primary cultures of residual embryonic *Jak1* KO DRG neurons do not survive when neuropoietic cytokines are used as a source of trophic support (4, 5). The broadly similar domain structure and substrate specificity of the JAKs suggest that non-redundant roles of JAK1 are likely due to differential regulation, perhaps by specific post-translational modification (PTM), but the molecular underpinnings of such regulation have been unclear.

With regard to potential JAK1-specific PTMs, it is intriguing that numerous unbiased proteomic studies suggest that JAK1 is modified by the lipid palmitate, while other JAK family members are likely palmitoylated far less frequently or not at all (6). However, the importance of palmitoylation for JAK1 signaling, especially in DRG neurons, has not been addressed.

Palmitoylation (also known as *S*-acylation) is best known to target proteins to specific membranes. However, unlike protein-lipid modifications such as myristoylation and farnesylation, which are restricted to protein N- and C-termini, respectively, palmitoylation can potentially occur at any position within a protein sequence containing a suitable cysteine residue. Palmitoylation can thus more readily impact additional aspects of protein interactions and, in the case of kinases, enzymatic activity (7, 8, 9, 10, 11). For example, we previously reported the surprising finding that palmitoylation is essential for the enzymatic activity, even in *in vitro* assays, of dual leucine-zipper kinase (DLK) (7). At that time, the lack of available reagents, such as phospho-specific antibodies, to probe DLK’s phosphorylation and activation state impacted our ability to define exactly how palmitoylation regulates DLK. However, we were aware that an array of well-characterized phospho-antibodies against JAKs might facilitate insights into palmitoylation-dependent control of JAK1 activity, should we observe it.

Here we report that JAK1 is robustly palmitoylated in transfected non-neuronal cells and endogenously in embryonic DRG neurons, and we identify the closely related protein acyltransferases (PATs), ZDHHC3 and ZDHHC7, as dominant regulators of JAK1 palmitoylation. At the molecular level, we reveal a critical role for palmitoylation in the control of JAK1 kinase activity, even *in vitro*, which is likely explained because palmitoylation is essential for the transphosphorylation of key sites in JAK1’s activation loop. We use available JAK1 cryoEM and AlphaFold2 structures to infer how palmitoylation may affect JAK1 conformation and facilitate transphosphorylation. Interestingly, this requirement for palmitoylation cannot be overcome by a heterologous lipid modification but is overridden by a well-known leukemia-associated *JAK1* mutation. Finally, we show that JAK1 palmitoylation is critical for DRG neuron signaling and survival driven by the neuropoietic cytokine LIF and, consistent with these findings, that *Zdhhc3/7* knockdown phenocopies loss of palmitoyl-JAK1 to blunt DRG neuron responses to LIF. These findings have considerable relevance for our understanding of JAK1 signaling in both physiological and pathological contexts and provide further insights into how palmitoylation can control not just localization, but also other key properties, of signaling enzymes.

## Results

### JAK1 palmitoylation is predominantly controlled by ZDHHC3 and ZDHHC7 in transfected cells and in cultured DRG neurons

**Figure 1:**
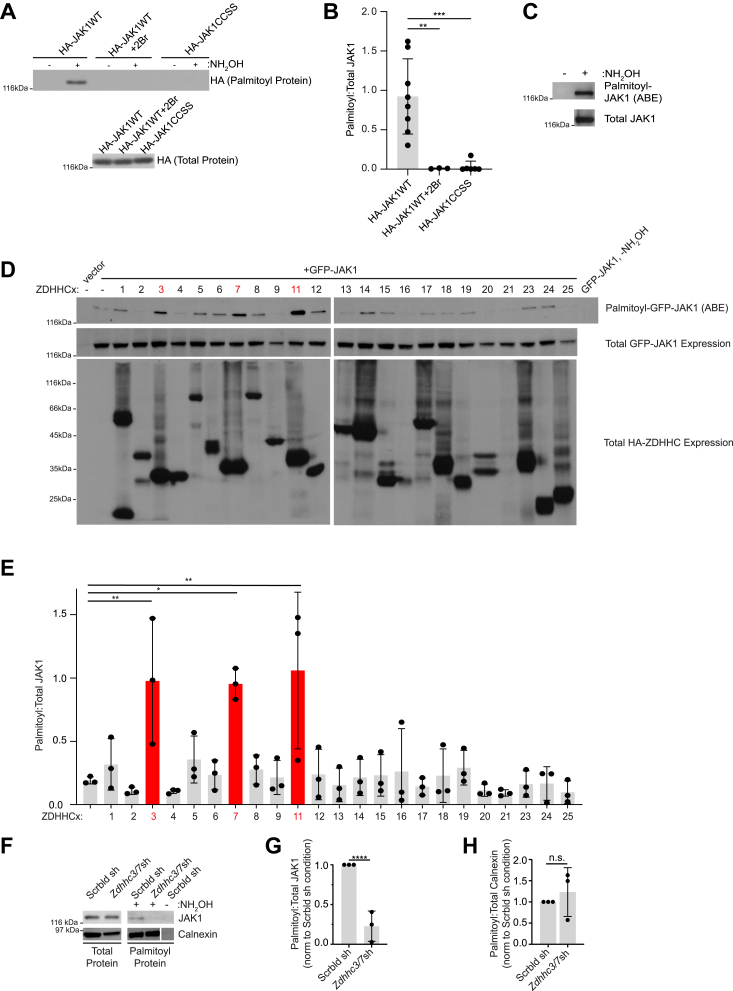
**JAK1 is palmitoylated in transfected HEK293T cells and in DRG neurons.***A*, Western blots of palmitoyl-fractions (isolated by ABE, *upper panel*) and total lysates (*lower panel*) of HEK293T cells transfected with the indicated JAK1 cDNA constructs and treated with or without 2-Bromopalmitate (2Br, 100 μM). *B*, quantified data from *A* confirm robust palmitoylation of JAK1WT that is prevented by 2Br treatment and by CCSS mutation. N = 3 to 7 per condition. ∗∗∗*p* < 0.001, ∗∗*p* < 0.01 *versus* HA-JAK1WT, one-way ANOVA with Dunnett *post hoc* test. F (2, 14) = 14.80, *p* = 0.0004. Data in this and all subsequent Figures are mean ± SD. *C*, Western blots to detect JAK1 in ABE fractions (*upper*) and total lysates (*lower*) from DRG neurons. Representative of three experiments. *D*, ABE fractions (*upper panels*) and total lysates (*middle*, *lower panels*) from HEK293T cells transfected to express GFP-JAK1 plus the indicated HA-ZDHHC PATs and blotted to detect GFP-JAK1 and HA-tagged ZDHHC-PATs. The number of samples analyzed in this experiment required samples for HA-ZDHHC1-12 (*left panels*) and HA-ZDHHC13 to 25 (*right panels*) to be loaded on two back-to-back gels, but resultant blots for each indicated antibody were processed side-by-side and *left* and *right panels* are from the same exposure. *E*, quantified data from *D* confirm that ZDHHC3, ZDHHC7, and ZDHHC11 most robustly palmitoylate JAK1. ∗∗*p* < 0.01, ∗*p* < 0.05 *versus* JAK1 alone condition, N = 3 to 4 determinations per condition, one-way ANOVA with Dunnett *post hoc* test. F (23, 48) = 5.116, *p* < 0.0001. *F*, total lysates (*left panels*) and ABE fractions (*right panels*) from DRG neurons, immunoblotted with the indicated antibodies. On the *lower right* Calnexin blot, the break indicates that intervening spacer lanes were removed, but all three lanes are from the same exposure. *G* and *H*, quantified data from *F* confirm that *Zdhhc3/7* knockdown greatly reduces endogenous JAK1 palmitoylation but does not affect calnexin palmitoylation (*G*: N = 3, Unpaired *t* test, *p* < 0.0001; *H*: N = 3, Unpaired *t* test, *p* = 0.5230).

As a first step to determine whether JAK1 is indeed palmitoylated, we expressed HA-tagged wild-type JAK1 (HA-JAK1WT) in HEK293T cells and performed a non-radioactive palmitoylation assay, Acyl Biotin Exchange (ABE; (12, 13)). We detected a strong signal for palmitoyl-HA-JAK1WT in ABE samples, which was absent when cells were pre-treated with a broad spectrum palmitoylation inhibitor, 2-bromopalmitate (2Br; (14)), or when ABE samples were prepared in the absence of the key reagent hydroxylamine (NH_2_OH)(Fig. 1, *A* and *B*). The palmitoyl-JAK1 signal was also abolished when two cysteines that lie in the linker region upstream of JAK1’s pseudokinase domain were mutated to non-palmitoylatable serine (Cys541, Cys542, ‘HA-JAK1CCSS’) (Fig. 1, *A* and *B*), consistent with (15). Together, these findings suggest that JAK1 is indeed palmitoylated and that Cys541 and Cys542 are the major JAK1 palmitoyl sites.

We next asked whether endogenous JAK1 is palmitoylated in cultured DRG neurons. We observed a strong, hydroxylamine-dependent signal for palmitoyl-JAK1 in ABE fractions from cultured DRG neurons (Fig. 1*C*), suggesting that JAK1 is endogenously palmitoylated in DRG neurons.

To identify PAT(s) for JAK1, we screened HA-tagged versions of all 23 mammalian PATs for their ability to palmitoylate co-expressed GFP-tagged JAK1WT (GFP-JAK1) in HEK293T cells. All HA-PATs were detectably expressed in our experiments, except for HA-ZDHHC21, which is often expressed at very low levels in studies using similar cDNA constructs (*e.g.* (16, 17, 18)). We found that three PATs, the related ZDHHC3 and ZDHHC7, and ZDHHC11, increased GFP-JAK1 palmitoylation to a greater extent than any other PAT (Fig. 1, *D* and *E*). Of these PATs, mRNA expression of *Zdhhc3* and *Zdhhc7* is reported to be markedly higher than that of *Zdhhc11* in DRG neurons ((19); www.mousebrain.org), so we assessed the importance of ZDHHC3 and ZDHHC7 for endogenous neuronal JAK1 palmitoylation. Palmitoylation of endogenous JAK1 was markedly reduced in ABE fractions from DRG neurons that had been lentivirally infected to express shRNAs that potently knock down *Zdhhc3* and *Zdhhc7* (“*Zdhhc3/7sh*”; Fig. 1, *F* and *G*; effectiveness of shRNAs confirmed in Fig. S1). In contrast, *Zdhhc3/7* knockdown did not affect the palmitoylation of calnexin, whose palmitoylation is ascribed to other PATs (20)(Fig. 1, *F* and *H*). These findings suggest that ZDHHC3/7 are essential for endogenous JAK1 palmitoylation in DRG neurons.

### Palmitoylation minimally affects JAK1 axonal localization in DRG neurons

**Figure 2:**
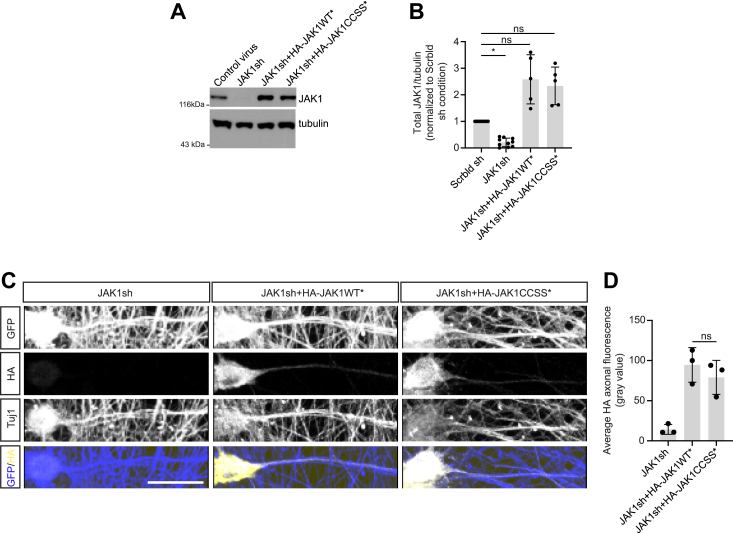
**Palmitoyl-site mutation minimally affects JAK1 axonal localization in cultured DRG neurons.***A*, Western blots, with the indicated antibodies, of lysates from cultured DRG neurons infected with lentiviruses to express GFP alone (first column), GFP and *Jak1* shRNA (second column), or GFP/*Jak1* shRNA plus shRNA-resistant (shr) HA-JAK1WT or HA-JAK1CCSS (third, fourth columns). *B*, Quantified data from *A* confirm effective knock down of endogenous Jak1 and rescue by shr-HA-JAK1WT and -HA-JAK1CCSS to levels that do not differ significantly from endogenous JAK1. ∗*p* < 0.05; ns: not significant, non-parametric one-way ANOVA with Dunn’s multiple comparison test, Kruskal-Wallis statistic 26.82, *p* < 0.0001. *C*, example images of DRG neurons lentivirally infected as in *A*, fixed and stained with the indicated antibodies. Tuj1 antibody recognizes neuron-specific beta-3 tubulin. *D*, quantified data of HA-positive axonal signal (3 biological replicates, total of 69–73 axons per condition) from C. Palmitoyl-site (CCSS) mutation does not significantly affect HA-JAK1 axonal localization, one-way ANOVA with Dunnett’s *post hoc* test. F (2, 6) = 17.09, *p* = 0.0033. Scale bar: 20 μm.

We next sought to understand the functional role(s) of JAK1 palmitoylation. Palmitoylation is perhaps best known to control protein trafficking and/or localization (11), and so we first assessed the extent to which palmitoylation affects JAK1 localization in DRG neurons, a cell type in which JAK1 is critically important (4). To minimize the likelihood that results were affected by JAK1 over-expression, we identified an effective *Jak1* shRNA, which we then lentivirally delivered to create a JAK1 “knockdown” background (Fig. 2*A*). We then co-infected *Jak1* knockdown neurons with viruses to express shRNA-resistant forms of either HA-JAK1WT or HA-JAK1CCSS (HA-JAK1WT∗, HA-JAK1CCSS∗). Western blots of lysates from DRG neurons cultured under these conditions confirmed that HA-JAK1WT∗ and HA-JAK1CCSS∗ expressed at similar levels to one another, close to levels of endogenous JAK1 (Fig. 2, *A* and *B*). Under these conditions, axonal localization of HA-JAK1WT and HA-JAK1CCSS was very similar (Fig. 2, *C* and *D*), suggesting that palmitoylation does not significantly affect JAK1 axonal localization in DRG neurons.

### Palmitoylation is essential for JAK1 kinase activity

**Figure 3:**
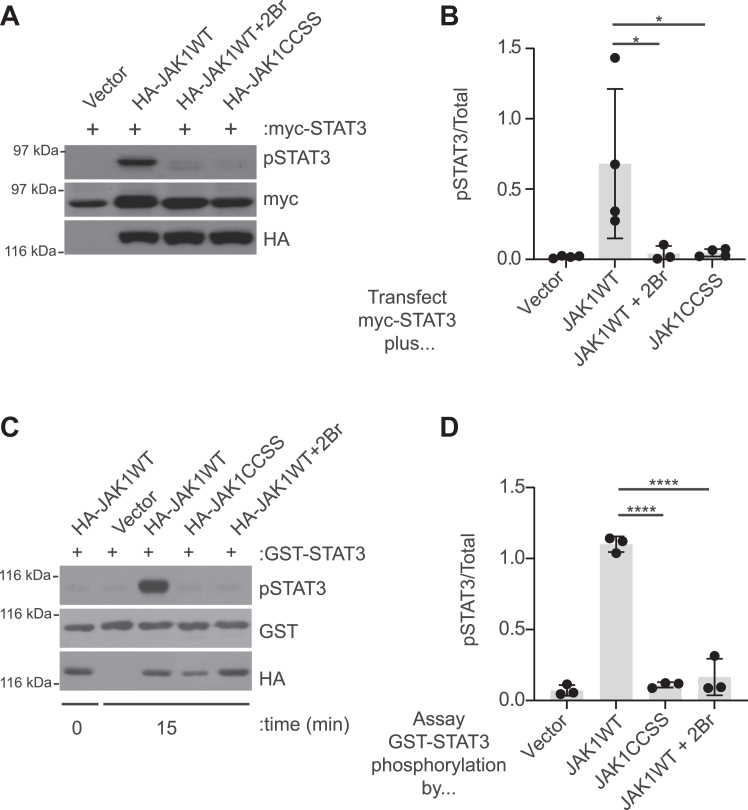
**Palmitoylation is essential for JAK1 to phosphorylate STAT3 in cotransfected HEK293T cells and *in vitro*.***A*, Western blots of lysates from HEK293T cells transfected with the indicated constructs and treated with or without 2Br prior to lysis. *B*, quantified data from *A* confirm that cotransfection of JAK1 greatly increases STAT3 phosphorylation, an effect prevented by 2Br treatment or CCSS mutation. ∗*p* < 0.05, one-way ANOVA with Dunnett *post hoc* test. N = 3 to 4 per condition. F (3, 11) = 5.259, *p* = 0.0171. *C*, *in vitro* kinase assays of HA immunoprecipitates from lysates expressing the indicated JAK1 constructs, using purified GST-STAT3 as substrate. Reactions were stopped at the indicated times. *D*, quantified data from *C* confirm robust phosphorylation of GST-STAT3 by JAK1WT, which is prevented by JAK1 CCSS mutation and by 2Br pretreatment of JAK1WT-expressing cells. ∗∗∗∗*p* < 0.0001, one-way ANOVA with Dunnett *post hoc* test. N = 3 per condition. *F* (3, 8) = 137.7, *p* < 0.0001.

The lack of effect of palmitoyl-site mutation on JAK1 axonal localization led us to ask if palmitoylation affects a different aspect of JAK1 function. We previously reported that palmitoylation is important for the enzymatic activity of DLK (7). In contrast to several other palmitoyl-kinases, DLK’s palmitoyl site lies in the “core” of its protein sequence, that is, away from the N- and C-termini, and might thus more readily affect this aspect of DLK function (21). Importantly, JAK1’s palmitoyl sites are also within the core of its sequence, so we asked if palmitoylation is necessary for JAK1 to phosphorylate its best-known substrate, Signal Transducer and Activator of Transcription-3 (STAT3) (22). Phosphorylation of myc-tagged STAT3 (myc-STAT3) at the key activatory site Y705 was undetectable in transfected HEK293T cells but was markedly increased by cotransfected HA-JAK1WT (Fig. 3, *A* and *B*). In contrast, myc-STAT3 phosphorylation at Y705 was minimally increased by HA-JAK1WT in cells that were treated with 2Br, or in cells cotransfected with HA-JAK1CCSS (Fig. 3, *A* and *B*). These results suggest that palmitoylation is required for JAK1 to directly phosphorylate STAT3 in cells.

Although striking, the palmitoylation-dependent phosphorylation of myc-STAT3 by co-expressed JAK1 (Fig. 3, *A* and *B*) could still potentially be explained by differential subcellular localization of JAK1WT and JAK1CCSS, rather than by a direct requirement of palmitoylation for JAK1 kinase activity. To distinguish between these possibilities, we assessed JAK1’s ability to phosphorylate purified STAT3 in an *in vitro* assay. HA-JAK1WT immunoprecipitated from transfected HEK293T cells robustly phosphorylated GST-tagged STAT3 (GST-STAT3) at Y705 *in vitro* (Fig. 3, *C* and *D*). In contrast, GST-STAT3 phosphorylation by HA-JAK1CCSS, or by HA-JAK1WT immunoprecipitated from cells pre-treated with 2Br, was almost undetectable (Fig. 3, *C* and *D*). These findings suggest that the palmitoylation dependence of STAT3 phosphorylation by JAK1 is more likely due to the effects of palmitoylation on JAK1’s catalytic activity, rather than on JAK1’s intracellular localization.

**Figure 4:**
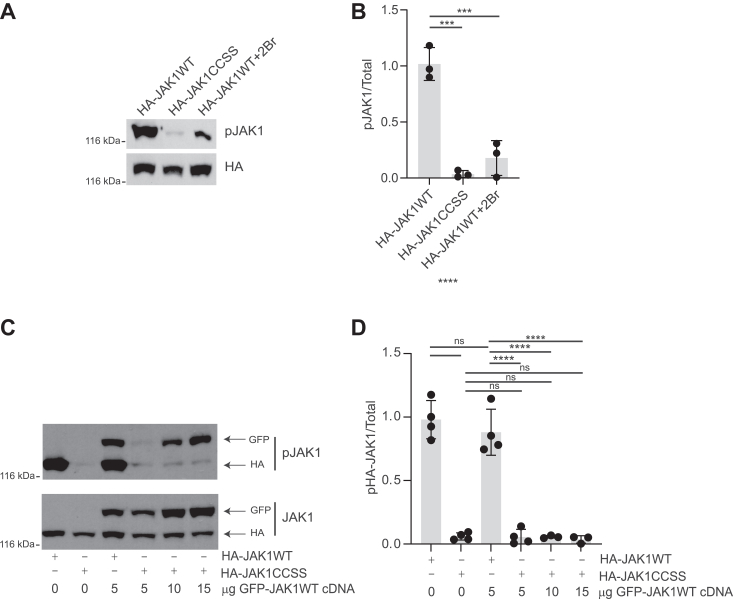
**Palmitoylation is required for transphosphorylation of activatory sites on JAK1.***A*, Western blots of HEK293T cell lysates transfected to express the indicated JAK1 constructs and treated with or without 2Br prior to lysis. *B*, quantified data from *A* confirm that CCSS mutation and 2Br treatment both greatly reduce JAK1 phosphorylation at Y1034/Y1035. ∗∗∗*p* < 0.001, one-way ANOVA with Dunnett *post hoc* test. N = 3 per condition. F (2, 6) = 54.20, *p* = 0.0001. *C*, Western blots from HEK293T cells transfected to express the indicated HA-tagged JAK1 constructs with or without the indicated amounts of GFP-tagged JAK1WT (GFP-JAK1WT). *D*, quantified data from *C* confirm that HA-JAK1 CCSS mutation prevents phosphorylation by GFP-JAK1WT, even when increased amounts of GFP-JAK1WT are expressed. ∗∗∗∗*p* < 0.0001, one-way ANOVA with Tukey *post hoc* test. N = 3 to 4 per condition. *F* (5, 16) = 69.40, *p* < 0.0001.

We therefore sought to gain more insight into why palmitoylation is necessary for JAK1’s ability to phosphorylate STAT3. To address this question, we used a phospho-specific antibody that detects phosphorylation of tyrosine sites in the activation loop of JAK1’s kinase domain (Y1034, Y1035) that are critical for the enzymatic activity of JAK1WT (23). HA-JAK1WT was robustly phosphorylated at Y1034/Y1035, but phosphorylation of these sites on HA-JAK1CCSS, or on HA-JAK1WT isolated from 2Br-treated cells, was far lower (Fig. 4, *A* and *B*). These results suggest that palmitoylation is essential for the phosphorylation of JAK1WT at the key activatory sites, Y1034/Y1035.

JAK1 is thought to become activated following transphosphorylation of Y1034/Y1035 within a JAK dimer (24, 25). At least two explanations could account for the weak phosphorylation of Y1034/Y1035 when JAK1CCSS is expressed alone (Fig. 4, *A* and *B*). First, CCSS mutation may render Y1034/Y1035 inaccessible or otherwise incapable of being phosphorylated (“unphosphorylatable”). Alternatively, JAK1CCSS might still be phosphorylatable at Y1034/Y1035 (and thus potentially “activatable”) if an active (WT) JAK1 were present to transphosphorylate these sites. To distinguish between these possibilities, we expressed HA-JAK1WT or HA-JAK1CCSS with GFP-JAK1WT. In lysates from cells co-expressing HA-JAK1WT and GFP-JAK1WT, we observed strong phospho-JAK1 Y1034/Y1035 signals corresponding to the predicted molecular weights of the HA- and GFP-tagged forms of JAK1 (Fig. 4*C* (lane 3), Fig 4*D*). In contrast, Y1034/Y1035 phosphorylation of HA-JAK1CCSS expressed alone was very weak and, moreover, was not increased by co-expressed GFP-JAK1WT (Fig. 4*C*, lanes 2 and 4, Fig. 4*D*). We also observed reduced Y1034/Y1035 phosphorylation of GFP-JAK1WT when co-expressed with HA-JAK1CCSS, compared to when co-expressed with HA-JAK1WT (Fig. 4*C*, lanes 3 and 4). We hypothesized that this reduced phosphorylation might be due to the formation of non-productive GFP-JAK1WT/HA-JAK1CCSS dimers in which the reduced activity of HA-JAK1CCSS precluded transphosphorylation of GFP-JAK1WT at Y1034/Y1035. Consistent with this explanation, increasing the ratio of GFP-JAK1WT:HA-JAK1CCSS effectively “rescued” the reduced GFP-JAK1WT phosphorylation (Fig. 4*C* lanes 4–6). However, even these higher amounts of GFPJAK1WT did not lead to phosphorylation of HA-JAK1CCSS (Fig. 4, *C* and *D*). These findings suggest that palmitoylation is critical for transphosphorylation of JAK1’s activatory sites, likely explaining why the loss of palmitoylation so markedly impacts the ability of JAK1WT to phosphorylate exogenous substrates.

### Structural insights into potential effects of JAK1 palmitoylation

We next asked whether existing structural information on JAK1 and AlphaFold2 models might provide insight into how palmitoylation controls JAK1 phosphorylation and activation. There is no experimental structure of JAK1 in the monomeric autoinhibited state containing both the pseudokinase and kinase domains, but there is a structure of a pseudokinase-kinase construct of the homologous protein TYK2 (PDB: 4OLI (26)). Recently, structures of homodimeric mouse JAK1 bound to a fragment Interferon lambda Receptor 1 (IFLR1) have appeared. In both structures, the JAK1 monomers dimerize primarily through head-to-head interactions of the N-terminal subdomains of the pseudokinase domains. However, in one structure (PDB: 7T6F (27), 3.6 Å resolution), the C-terminal active kinase domains of JAK1 are far apart, with the active sites facing away from each other. We refer to this form as the “substrate-competent dimer.” In the other (PDB: 8EWY (28), 5.5 Å resolution), the active kinase domains are face to face, and resemble the structures of autophosphorylation complexes of the activation loop in which the G-helices of the two monomers make contact with each other (28). In this form, an exogenous substrate would not be able to bind to the kinase active site. We refer to this structure as the “autophosphorylation dimer.”

**Figure 5:**
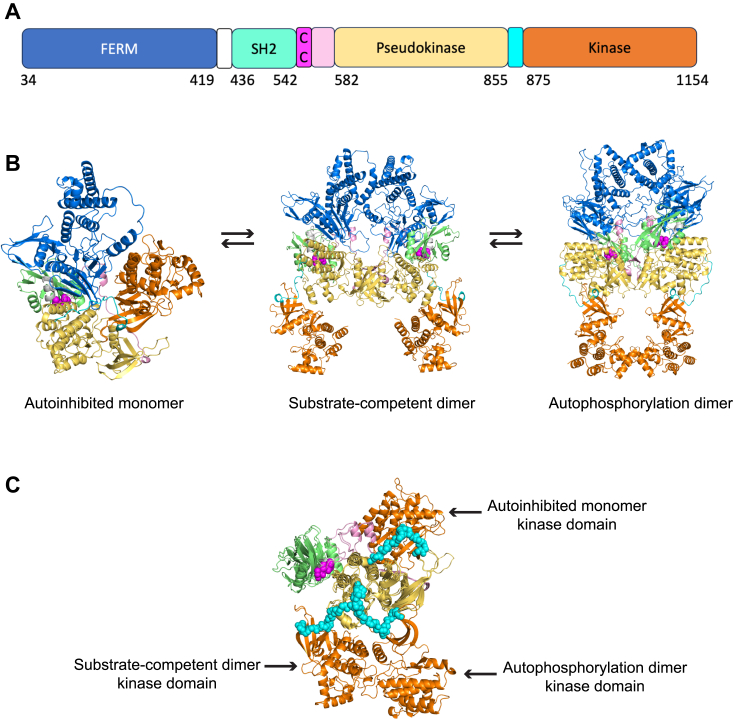
**Insights into potential effects of palmitoylation from AlphaFold2 models of human JAK1 in monomeric and dimeric forms.***A*, color scheme and residue boundaries for folded domains in JAK1 (length of bars not to scale). Linker regions are unlabeled between each pair of domains. *B*, potential equilibrium between autoinhibited monomeric human JAK1 model (*left*), substrate-competent homodimer of JAK1 (*center*), and autophosphorylation dimer of JAK1 (*right*). The order of states in the equilibrium may be different and could also involve partial transitions (*e.g.*, a dimer with one monomer in the autophosphorylation state and one in the substrate-competent state). *C*, superposition of monomers from autoinhibited monomer model, substrate-competent homodimer model, and autophosphorylation dimer model. Domains are colored as in *A* and *B*. The FERM domain is not shown for clarity. The linker between the pseudokinase and kinase domain (backbone atoms in *cyan spheres*, residues 856–874) undergoes a large motion, placing the kinase domain in three different positions relative to the relatively fixed relative orientations of the FERM, SH2, and pseudokinase domains. Note the proximity of the palmitoyl sites (*magenta*) to the apparent fulcrum for rotation of the pseudokinase-kinase linker.

To obtain structural information on human JAK1, we used AlphaFold2 and AlphaFold-Multimer (v3) (without the use of templates) to make models of full-length monomeric and homodimeric human JAK1. The top-scoring models (of 20) of the monomeric protein have an arrangement of the pseudokinase and kinase domains similar to that of the autophosphorylation dimers of mouse JAK1 (PDB: 8EWY). However, several of the lower-scoring models match the arrangement in the TYK2 autoinhibited structure (PDB: 4OLI). In our models of autoinhibited human JAK1 (color-coded according to JAK1 domain structure; Fig. 5, *A* and *B*), the SH2 domain is immediately adjacent to the C-terminal domain of the pseudokinase. Cys541-Cys542 (magenta spheres), which are at the very end of the SH2 domain, is about 8 Å away from residues in the loop between the H and I helices of the pseudokinase domain (residues 829–832).

AlphaFold-Multimer (v3; (29)) made distinct models of human JAK1 that strikingly resemble either the substrate-competent dimer (PDB: 7T6F) or the autophosphorylating dimer (PDB:8EWY). These are shown in the center and right side of Figure 5*B*, respectively. We note that AlphaFold-Multimer (v3) was trained on data in the PDB released through September 2021 and thus did not have either of the two mouse JAK1 homodimer structures in its training data. In both the monomer and dimer structures, the FERM, SH2, and pseudokinase domains have the same arrangement relative to one another. The palmitoyl-sites Cys541-Cys542 are not in direct contact with the linker between the pseudokinase and active kinase domains (residues 856–874), which undergoes significant repositioning when going from one state to another (Fig. 5*C*). However, they are in contact with the C-terminal domain of the pseudokinase, which binds the linker directly *via* the G-helix and loops connecting it to the F and H helices. This proximity to the linker, particularly to the apparent fulcrum for repositioning between the three states, raises the possibility that palmitoylation of Cys541-Cys542 influences which state is favored. We consider the implications of these structures further in the Discussion section.

### A *JAK1* oncogenic mutation can compensate for palmitoylation but a heterologous lipid cannot

**Figure 6:**
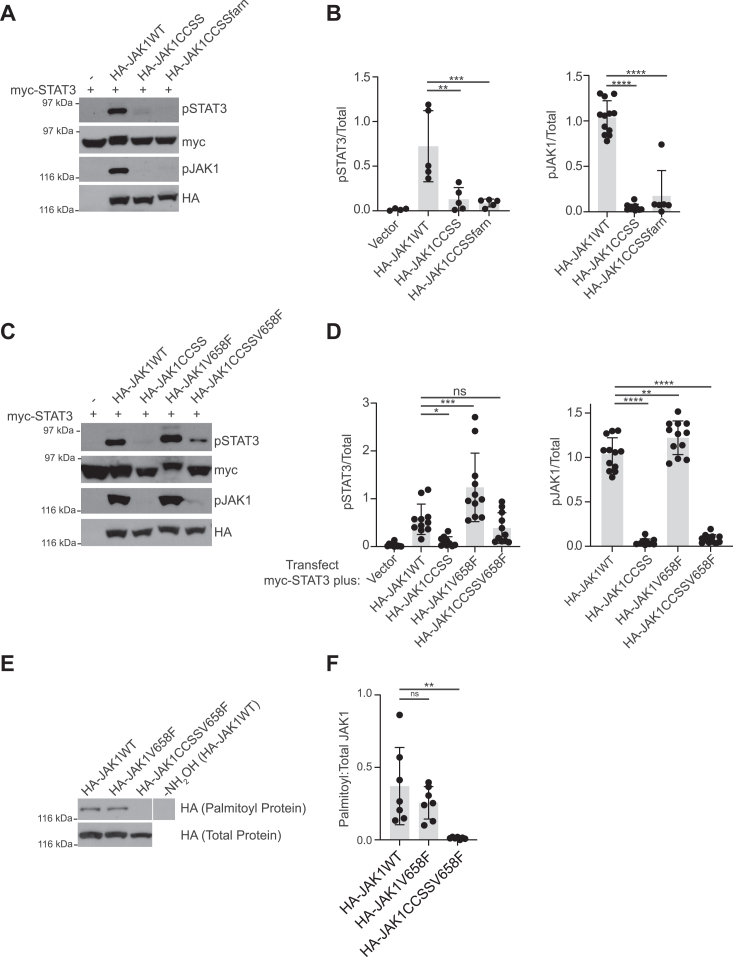
**Palmitoylation-dependence of JAK1 activity is overridden by oncogenic mutation but not by farnesylation.***A*, Western blot of HEK293T cell lysates transfected with myc-STAT3 and indicated JAK1 constructs. *B*, quantified data from *A* confirm that JAK1CCSS mutation greatly impairs phosphorylation of STAT3 (*left panel*, N = 4–5 per condition, one-way ANOVA with Dunnett *post hoc* test, ∗∗∗*p* < 0.001, ∗∗*p* < 0.01 and JAK1 (*right panel*, N = 6 per condition, one-way ANOVA with Dunnett *post hoc* test, ∗∗∗∗*p* < 0.0001), and that neither STAT3 nor JAK1 phosphorylation can be rescued by farnesylated JAK1 (JAK1CCSSfarn). F (3, 15) = 10.84, *p* = 0.0005 for pSTAT3. F (2, 26) = 107.1, *p* < 0.0001 for pJAK1. *C*, Western blot of HEK293T cell lysates transfected with myc-STAT3 and indicated JAK1 constructs. *D*, *left panel*: Quantified data from *C* confirm increased STAT3 phosphorylation by JAK1V658F and similar STAT3 phosphorylation by JAK1WT and JAK1CCSSV658F. N = 10 to 11 per condition, one-way ANOVA with Dunnett *post hoc* test, ∗∗∗*p* < 0.001, ∗*p* < 0.05. *F* (4, 48) = 16.35, *p* < 0.0001. *Right panel*: Despite its ability to phosphorylate STAT3, phosphorylation of JAK1CCSSV658F at Y1034/Y1035 is minimal. N= 11 to 12 per condition, one-way ANOVA with Dunnett *post hoc* test, ∗∗∗∗*p* < 0.0001, ∗∗*p* < 0.01. *F* (3, 43) = 241.2, *p* < 0.0001. *E*, Western blot of ABE fractions (*top*) and total lysates (*bottom*) from HEK293T cells transfected to express the indicated JAK1 constructs. On the *upper* blot, the break indicates that intervening spacer lanes were removed, but all four lanes are from the same exposure. *F*, quantified data from *E* confirms similar palmitoylation levels of JAK1WT and JAK1V658F, but undetectable palmitoylation of JAK1CCSSV658F. ∗∗*p* < 0.01, one-way ANOVA with Dunnett *post hoc* test. N = 7 per condition. *F* (2, 18), *p* = 0.0026.

We next sought to determine whether other mechanisms and/or modifications might restore JAK1’s kinase activity in the absence of palmitoylation. A requirement for palmitoylation can sometimes be overcome by directing the addition of a heterologous lipid (13, 30, 31, 32), so we first assessed phosphorylation of STAT3 by a JAK1CCSS mutant carrying an additional consensus for the addition of a farnesyl lipid (HA-JAK1CCSS-farn). However, HA-JAK1CCSS-farn was unable to phosphorylate cotransfected STAT3 and also showed no phosphorylation at Y1034/1035 (Fig. 6, *A* and *B*). These results suggest that a heterologous farnesyl lipid cannot substitute for palmitoylation to restore JAK1 kinase activity.

We also asked whether JAK1 V658F mutation, which was identified in cases of leukemia (33) and is reported to increase JAK1 activity in cells (34), can overcome the requirement for palmitoylation. V658F mutation indeed increased JAK1-dependent phosphorylation of STAT3 at Y705 (Fig. 6, *C* and *D*), consistent with (34). V658F mutation also increased JAK1 phosphorylation at Y1034/1035 (Fig. 6, *C* and *D*). Importantly, the ability of an HA-JAK1CCSS-V658F compound mutant to phosphorylate STAT3 at Y705 did not differ significantly from that of HA-JAK1WT, although the compound mutant was not detectably transphosphorylated at Y1034/1035 (Fig. 6, *C* and *D*). We asked whether palmitoylation of HA-JAK1CCSS-V658F at non-physiological sites might account for this result, but HA-JAK1CCSS-V658F was not detectably palmitoylated, rendering this explanation unlikely (Fig. 6, *E* and *F*). Together, these findings suggest that V658F mutation can circumvent the requirement for palmitoylation, facilitating JAK1-dependent phosphorylation of exogenous substrates.

### Palmitoylation by ZDHHC3/7 is important for JAK1-dependent signaling and survival of DRG neurons

**Figure 7:**
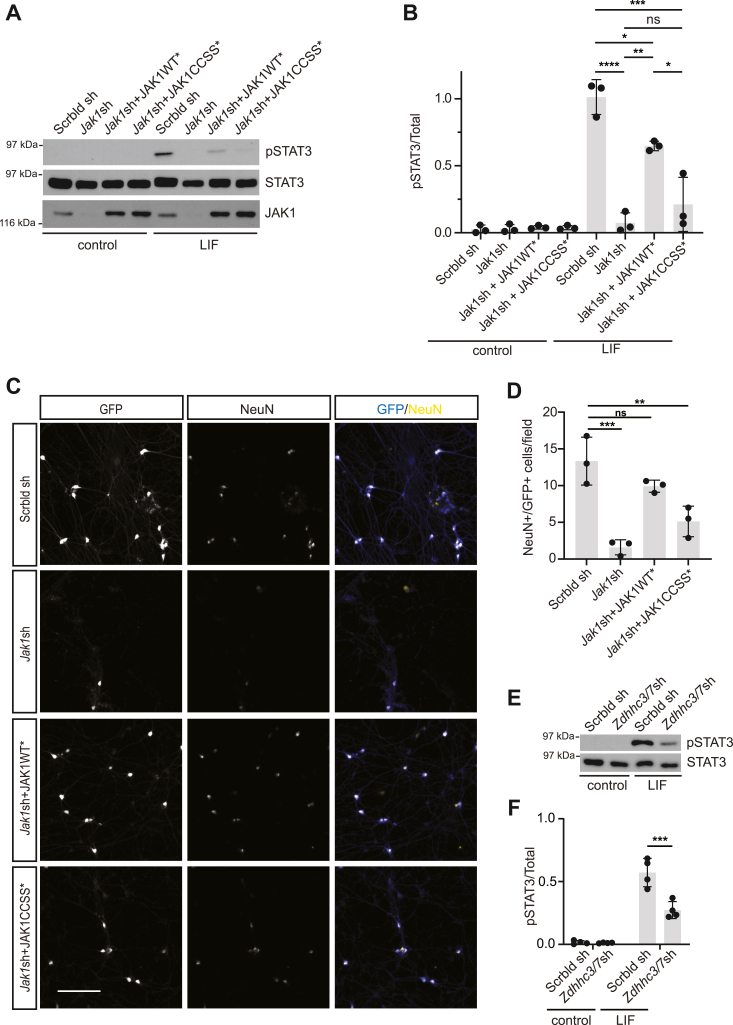
**JAK1 palmitoylation by ZDHHC3/7 is important for DRG neuron signaling and survival in response to neuropoietic cytokines.***A*, Western blots of lysates of DRG neurons that had been cultured in the continued presence of NGF to ensure survival, infected with the indicated lentiviruses and subsequently stimulated with LIF. *B*, quantified data from *A* confirm robust LIF-induced STAT3 phosphorylation, which is prevented by *Jak1* knockdown and rescued by JAK1WT∗ but not JAK1CCSS∗. ∗∗∗∗*p* < 0.0001; ∗∗∗*p* < 0.001; ∗∗*p* < 0.01; ∗*p* < 0.05, one-way ANOVA with Tukey test. N = 3 per condition. F (3, 8) = 33.91, *p* < 0.0001. *C*, images of cultured DRG neurons immunostained with the indicated antibodies 4 days after being plated in the presence of LIF and infected with lentiviruses to express GFP plus a scrambled shRNA (Scrbld sh; *top row*), GFP plus *Jak1* shRNA (Jak1sh; second row), GFP, *JAK1* shRNA and shRNA-resistant JAK1WT (JAK1WT∗; third row) or GFP, *JAK1* shRNA and shRNA-resistant JAK1CCSS (JAK1CCSS∗; fourth row). Scale bar: 100 μm. *D*, quantified data from *C* confirm greatly reduced numbers of GFP^+^/NeuN^+^ neurons after *Jak1* knockdown, an effect rescued by JAK1WT∗ but not JAK1CCSS∗. ∗∗∗*p* < 0.001, ∗∗*p* < 0.01, one-way ANOVA with Dunnett *post hoc* test. N = 3 per condition. *F* (3, 8) = 19.19, *p* = 0.0005. *E*, Western blots from control (expressing scrambled sh; Scrbld sh) or *Zdhhc3/7* knockdown DRG neurons stimulated with LIF or left unstimulated and immunoblotted with the indicated antibodies. *F*, quantified data from *E* confirm reduced LIF-induced STAT3 phosphorylation in *Zdhhc3/*7 knockdown neurons. ∗∗∗*p* < 0.001, two-way ANOVA with Tukey test. N = 4 per condition. Virus condition *p* < 0.001 (*F* (1, 14) = 17.28), treatment *p* < 0.0001 (*F* (1, 14) = 122.8), interaction *p* = 0.0012, (*F* (1, 14) = 16.34).

Finally, we assessed the functional importance of palmitoylation for physiological JAK1 signaling in neurons. To address this question, we cultured DRG neurons in the presence of Nerve Growth Factor (NGF) to maintain neuron viability, stimulated cultures with LIF, and assessed phosphorylation of STAT3. We observed robust LIF-induced phosphorylation of STAT3 in DRG neurons infected with lentivirus expressing a control scrambled shRNA, but LIF-induced STAT3 phosphorylation was almost undetectable in *Jak1* “knockdown” DRG neurons (Fig. 7, *A* and *B*). We next assessed the ability of shRNA-resistant HA-JAK1WT or HA-JAK1CCSS to rescue LIF-induced STAT3 phosphorylation in *Jak1* knockdown neurons. LIF-induced STAT3 phosphorylation was significantly rescued in DRG neurons expressing shRNA-resistant HA-JAK1WT but remained impaired in neurons expressing shRNA-resistant HA-JAK1CCSS (Fig. 7, *A* and *B*). Phosphorylation of STAT3 in neurons exposed to Ciliary Neurotrophic Factor (CNTF, another neuropoietic cytokine that potently activates JAK-STAT signaling (4)) was also palmitoyl-JAK1-dependent (Fig. S2). The efficacy of *Jak1* knockdown and rescue for these studies was confirmed as in Figure 2*A*. Together, these findings suggest that palmitoylation is important for JAK1-dependent signaling in DRG neurons.

JAK1 is important for DRG neuron viability when LIF is used as a sole source of trophic support (4), so we asked whether this role of JAK1 requires its palmitoylation. Lentiviral infection with *Jak1* shRNA, but not a control scrambled shRNA, markedly reduced survival of DRG neurons cultured in the presence of LIF (Fig. 7, *C* and *D*). This finding is reminiscent of the impaired survival of DRG neurons cultured from *Jak1* KO mice in the presence of LIF (4) and further suggests that reduced DRG neuron survival in the absence of JAK1 *in vivo* is due to an autonomous requirement for JAK1 in these cells. Importantly, similar to LIF-induced STAT3 phosphorylation, DRG neuron survival in the presence of LIF was rescued by coinfected HA-JAK1WT but not by HA-JAK1CCSS (Fig. 7, *C* and *D*). These findings suggest that JAK1 palmitoylation is important for LIF-induced DRG neuron survival.

Finally, we asked if loss of *Zdhhc3/7*, the PATs that dominantly control JAK1 palmitoylation in neurons (Fig. 1), phenocopies loss of palmitoyl-JAK1. Indeed, LIF-induced phosphorylation of STAT3, which is palmitoyl-JAK1-dependent (Fig. 7*A*), was significantly impaired in *Zdhhc3/7* knockdown DRG neurons (Fig. 7, *E* and *F*). We also found a similar reduction in LIF-induced STAT3 phosphorylation in neurons infected to express a second pair of *Zdhhc3/7* shRNAs (Figs. S1 and S3). These findings are consistent with a model in which ZDHHC3/7 dominantly control endogenous JAK1 palmitoylation.

## Discussion

Although JAK1 was previously identified as a likely palmitoylated kinase (6, 15), whether and how this modification impacts endogenous physiological and/or pathological signaling by JAK1 has not been addressed. One major finding of this study is that palmitoylation of JAK1, controlled by the related PATs ZDHHC3 and ZDHHC7, is essential for JAK1’s roles in LIF-dependent signaling and survival of DRG sensory neurons (Fig. 7).

Our findings also help clarify how JAK1 signaling controls the development of DRG neurons themselves. JAK1 signaling, which depends on the upstream transmembrane protein Gp130, is critical for the survival of DRG neurons supported by neuropoietic cytokines in culture (4, 5). Gp130-JAK1 signaling is also critical for DRG neuron survival *in vivo*, given the reduced number of DRG neurons found in, or isolated from, conventional *Gp130* or *Jak1* KO mice (4, 5). Importantly, the time window during which Gp130/JAK signaling is critical *in vivo* (approx. E14-E17 for mice; (5), likely equivalent to E16-E20 in rats (35)) is highly consistent with our findings from cultured neurons, which focus on neurons from E16 rats for their initial 4 days in culture. It is of note that the *in vivo* time window for this requirement is likely brief, because conditional knockout of either *Gp130* (gene name *IL6st*) or *Jak1* with Nav1.8-Cre, which is active perinatally, has little effect on DRG neuron survival (36, 37). This result suggests that, just as DRG neurons expressing the NGF receptor *TrkA* become NGF-independent during later development (38), the neuropoietic cytokine-dependent pool of DRG neurons rapidly becomes cytokine-independent for survival and/or that other trophic factors can substitute for neuropoietic cytokines at these later time points. Nonetheless, there is a critical time window during which gp130/palmitoyl-JAK1 signaling is essential for the survival of a significant subset of DRG neurons.

In seeking to define the importance of palmitoyl-JAK1 signaling, we focused on STAT3, the best described JAK substrate, which (i) mediates a wide variety of ‘downstream’ JAK-dependent events and (ii) causes serious deficits, including the death of neuropoietic cytokine-dependent sensory neurons both in culture and *in vivo*, when knocked out post-gastrulation (39). Consistent with STAT3 being a key palmitoyl-JAK1 substrate, rescue of LIF-induced STAT3 phosphorylation and LIF-dependent DRG neuron survival by JAK1WT, compared to JAK1CCSS, is very similar (Fig. 7). However, other substrates of palmitoyl-JAK1 may also contribute to DRG neuron survival.

While we focused our study on the neurodevelopmental roles of JAK1 palmitoylation, our findings may also increase understanding of JAK1 function in pathological conditions in adults. In particular, JAK1 expression in mature DRGs is particularly high in pruriceptive neurons that are implicated in chronic itch, and perinatal *Jak1* deletion in DRG neurons markedly reduces chronic itch without broadly affecting neuronal function (36). Based on our own findings in DRG neurons (Fig. 7), it is tempting to speculate that JAK1’s roles in chronic itch may also require its palmitoylation. Moreover, although pharmacological JAK inhibition reduces chronic itch *in vivo* (36), many JAK inhibitors do not distinguish between JAK1 (which is highly palmitoylated) and other JAK family members (which are likely palmitoylated only at low levels, if at all, but which play key roles in other cell types). It is thus an intriguing possibility that pharmacologically targeting JAK1 palmitoylation, rather than broadly inhibiting JAK kinase activity, could provide a means to alleviate chronic itch without affecting other physiological roles of JAK signaling.

It is also interesting that ZDHHC3/7 are the dominant PATs controlling JAK1 palmitoylation (Fig. 1). These PATs can palmitoylate an array of substrates (40, 41, 42, 43). What is therefore surprising is not, perhaps, that ZDHHC3/7 dominantly palmitoylate JAK1 in cells, but that the contribution of other PATs, especially those active towards JAK1 when overexpressed (Fig. 1, *D* and *E*), is apparently minor. Notably, both the axonal protein GAP-43 and the GABA receptor gamma2 subunit have been identified as ZDHHC3/7 substrates *in vivo* (40). Both these proteins contain a di-cysteine palmitoyl-motif that resembles that of JAK1, suggesting that ZDHHC3/7 effectively palmitoylates motifs of this type. It is also intriguing that *Jak1* KO, *Gp130* (*Il6st*) KO and *Zdhhc3/7* DKO mice all die perinatally (4, 5, 40), with those mice that have been examined thus far exhibiting a marked loss of DRG sensory neurons. There are potentially many reasons for the similarity of these findings, but it is an intriguing possibility that the presence of all these proteins in a common palmitoylation-dependent pathway accounts for this phenotypic overlap.

One of the most striking findings of this study is that palmitoylation exerts little effect on the localization of JAK1 in DRG neurons but is nonetheless critical for JAK1-dependent signaling, even *in vitro* (Figs. 2 and 3). We cannot exclude the possibility that palmitoylation affects the localization of a specialized sub-pool of JAK1 and/or is important for nano- but not micro-scale JAK1 localization, which might not be detected in our confocal imaging studies. However, the most parsimonious explanation for these findings is that palmitoylation predominantly affects JAK1’s kinase activity in neurons rather than its localization. JAK1 thus joins a short list of kinases, including Casein Kinase 1 (CK1) gamma, Fyn, Lck, LIM kinase-1 (LIMK1), and DLK, whose intracellular signaling critically requires this modification (21, 44). Notably, these palmitoyl-kinases fall into two distinct groups; for Fyn, Lck, LIMK1, and CK1gamma, palmitoylation is important for cellular function, but not essential for intrinsic catalytic activity assayed *in vitro* and thus appears to act predominantly as a determinant of kinase localization (8, 21, 44). In contrast, palmitoyl-mutant forms of DLK and JAK1 not only signal ineffectively in cells but also show greatly reduced kinase activity *in vitro* (Fig. 3 and (7)). Why might palmitoylation exert this unexpected effect on DLK and JAK1 activity, but not other palmitoyl-kinases? One possible explanation is that DLK and JAK1 are palmitoylated within the core of their protein sequences, while Fyn, Lck, LIMK1, and CK1gamma are all palmitoylated close to their N- or C-termini. In our prior studies on DLK, we suggested that palmitate addition to DLK’s “core” might have a greater ability to alter protein–protein interactions and/or domain structure and hence more directly affect intrinsic catalytic activity (7, 21). However, no reagents (such as phospho-specific antibodies) were available to assess DLK’s activation state and further test this hypothesis. Such antibodies are available for JAK1, and reveal that palmitoylation is critical for JAK1 transphosphorylation at Y1034/Y1035, key sites in the activation loop of JAK1’s kinase domain (23)(Fig. 4). We speculate that palmitoylation thus alters the structure and/or local environment of JAK1 to render these activatory sites more accessible, and/or to increase the intrinsic enzymatic activity of the JAK1 kinase domain, thereby coupling palmitoylation to JAK1’s catalytic activity.

Another intriguing finding is that the oncogenic V658F mutation largely overrides the requirement for palmitoylation to permit phosphorylation of STAT3 by JAK1 (Fig. 6, *D*–*F*). Because palmitoylation increases protein affinity for membranes, we previously proposed that palmitoylation may act as a “security feature” to ensure that key kinases are only active in a specific subcellular location (*e.g.* only on transport vesicles for DLK, and only close to the dendritic spine membrane for LIMK1 (21)). Our findings suggest that V658F mutation not only increases JAK1 activity in cells but also generates a form of JAK1 whose localization and activity are potentially uncoupled. Such a mutation would thus allow JAK1 to be active in non-physiological locations, where it might have far greater potential to phosphorylate inappropriate substrates and drive oncogenic changes. This hypothesis is worthy of further experimental investigation.

It is likewise interesting that JAK1CCSS-V658F is active towards STAT3 without being detectably phosphorylated at Y1034/Y1035 (Fig. 6, *C*-*D*). This finding is not without precedent, because both the WT form of another JAK, JAK3, and a chimera consisting of JAK3’s N-terminus fused to the pseudokinase and kinase domain of JAK1, can phosphorylate STAT family substrates, even when their activation loop tyrosines (homologous to JAK1 Y1034/Y1035) are mutated (23). Taken together with a report that V658F mutation does not alter JAK1’s enzymatic activity *in vitro* (45), these findings are consistent with a model in which V658F mutation affects JAK1 signaling in cells in a manner independent of JAK1’s activation loop. Consistent with this notion, a crystallographic study suggested that one key effect of V658F mutation is to alter the structure of JAK1’s pseudokinase domain and its upstream linker region (46). Intriguingly, F575, the residue whose position is most affected by V658F mutation (46), lies close within JAK1’s tertiary structure to the palmitoyl-sites (C542, C543), which also lie within the linker region. Together, these findings suggest that changes in the structure and/or environment of this linker can affect JAK1 function in two distinct ways; palmitoylation at C542/543 directly impacts JAK1 kinase activity by altering activation loop accessibility, whereas V658F mutation impacts F575 to affect pseudokinase-kinase domain interplay. The latter mechanism can thus override the requirement for activation loop phosphorylation of JAK1 which is normally critically dependent upon palmitoylation.

JAK1 structures (Fig. 5) provide additional insights into this model. Structures of JAK1 dimers suggest that one conformation is primarily substrate-competent, and another autophosphorylation-competent. Palmitoylation may encourage autophosphorylation by shifting the equilibrium from a predominantly substrate-competent configuration to a more equal balance between the two. In this way, JAK1 can be autophosphorylated and return to the substrate-competent form as an active kinase, accessible to the substrate. JAK1-CCSS is not detectably autophosphorylated and cannot be autophosphorylated by JAK1WT (Fig. 3). Our hypothesis is consistent with these data: a CCSS mutant does not easily move to the autophosphorylating configuration and is stuck in the substrate-competent position as an inactive, unphosphorylated kinase. Since both monomers must move to the autophosphorylating position, even JAK1-CCSS/WT heterodimers cannot autophosphorylate, which is only possible in WT homodimers. It is also possible that lack of palmitoylation shifts the equilibrium toward the monomeric state, which is unlikely to be autophosphorylated.

The available structural models cannot fully explain how palmitoylation affects JAK1, though. In particular, the increase in local hydrophobicity caused by palmitoylation would likely cause this region of JAK1 to become closely associated with the lipid membrane, with palmitate being inserted into the bilayer. The conformational change associated with this rearrangement would be predicted to significantly alter the spatial relationship of the different JAK1 domains, potentially facilitating JAK1 activation in additional ways.

One final factor to consider is the known interactions of JAK1 with other proteins. Given that palmitoylation does not affect JAK1 axonal enrichment (Fig. 2), it seems possible that protein-protein interactions, rather than palmitoylation per se, are the primary determinant of JAK1 localization in neurons. It might thus be necessary to consider multi-protein complexes containing not only JAK1 but also its upstream regulators and/or downstream substrates in order to fully define how palmitoylation regulates JAK1.

With these potential complementary mechanisms in mind, we recognize that the requirement for JAK1 palmitoylation for DRG neuron survival is less striking than that observed for JAK1 signaling in non-neuronal cells (compare Figs. 3 and 4 with Fig. 7). While these differences could be due to the different readouts assessed, DRG neurons may find alternate ways, perhaps akin to those observed for the V658F mutation (Fig. 6, *D*–*F*) and/or involving additional binding partners, to partially override the requirement for palmitoylation of JAK1 and thus ensure their survival. In this regard, it is interesting that even JAK1WT can undergo the conformational change in the pseudokinase domain that is seen in the JAK1 V658F mutant (46). An alternative explanation for the reduced palmitoyl dependence of JAK1 readouts in neuronal assays could be the presence of another JAK family member. However, the low expression of other JAKs in DRG neurons ((47) and mousebrain.org), and the likely minimal palmitoylation of these other JAKs (6), suggests that other explanations, such as JAK1 protein–protein interactions that partially overcome the requirement for palmitoylation, are more likely.

In this study, the availability of key reagents and published structures allowed us to gain greater insight into how palmitoylation regulates JAK1 signaling, compared to our prior work on palmitoylation of LIMK1 and DLK (7, 8). Importantly, previously generated reagents and structural data may facilitate studies of other palmitoyl-kinases, leading to further insights into how this lipid modification controls kinase signaling in diverse cell types and biological contexts.

## Experimental procedures

### Antibodies

The following antibodies, from the indicated sources, were used: rabbit anti-phospho-STAT3 (#9145), rabbit anti-STAT3 (#8768), rabbit anti-phospho-JAK1 (#74129), rabbit anti-myc (#9145), alpha-tubulin (DM1A, #3873) all from Cell Signaling Technology; mouse anti HA11 (#901514) and anti-Tuj1 (both from Biolegend); mouse anti-myc 9E10 (UPenn Cell Center); rabbit anti-GFP #A11122 (Invitrogen); mouse anti-JAK1 #610231 (BD Transduction Labs), mouse-anti NeuN #MAB377 (Millipore Sigma), Calnexin #10286 (Abcam). Unless otherwise noted, all chemicals were from ThermoFisher Biosciences and were of the highest reagent grade available.

### HEK293T cell culture and transfection

HEK293T cells were cultured in Dulbecco’s Modified Eagle Medium (DMEM, Thermo Fisher Scientific) with 10% fetal bovine serum (FBS; Hyclone), 1% Penicillin-Streptomycin (Thermo Fisher Scientific), and 1x GlutaMAX (Thermo Fisher Scientific). A calcium phosphate method described previously (48) was used for all HEK293T cell transfections.

### DRG Neuron culture

Timed-pregnant female Sprague Dawley rats (Strain code 400), Charles River) were euthanized at E16 and used for the dissociation of embryonic DRG neurons as previously described (7). A single-timed-pregnant rat was used per dissection and tissue from embryos of both sexes was pooled. All procedures involving animals followed National Institutes of Health guidelines and were approved by the Institutional Animal Care and Use Committee (IACUC) of Temple University. Conventional DRG “mass” cultures were plated in Neurobasal medium containing B27, GlutaMAX and 25 ng/ml NGF (Alomone Labs), plus Fluorodeoxyuridine to inhibit the growth of mitotic cells, as described (7). In some experiments, 25 ng/ml LIF (Alomone Labs) was used as trophic support rather than NGF. Neurons were re-fed at 1 day *in vitro* (DIV1) with the same medium (containing either NGF or LIF) as used for plating. For studies of neuronal survival, LIF-supported cultures were infected with lentiviruses on DIV1 and fixed on DIV4. For studies of JAK1 signaling and localization, NGF-supported cultures were infected with lentiviruses on DIV3 and stimulated with either 1 ng/ml (final concentration) LIF or with 10 pg/ml CNTF on DIV6 and were then lysed in buffer containing 4 volumes of Immunoprecipitation buffer (PBS containing 1 mM EDTA, 1 mM EGTA, 50 mM sodium fluoride, 5 mM sodium pyrophosphate, 1% (w/v) Triton X-100, 1 mM sodium orthovanadate, pH 7.4) mixed with 1 volume of 5x SDS sample buffer, plus freshly added 1% (v/v) betamercaptoethanol, 1 μM Microcystin-LR (Cayman Chemicals) and 1x Protease Inhibitor Cocktail (Roche).

### Lentiviral vectors and preparation

A *Jak1* shRNA (5′-gcctgagagtggaggtaac-3′) was identified bioinformatically and synthesized with a neighboring H1 promoter by Genewiz. The resultant cassette was subcloned into lentiviral vector FEGW (Holland *et al.*, 2016), as was a homologous cassette containing a scrambled shRNA (original shRNA sequence from Origene). Human *JAK1* cDNA was obtained from DNASU and subcloned into HA-FEW or GFP-FEW vectors using primers with *XhoI* and *NotI* restriction sites. Human *JAK1* cDNA was made shRNA-resistant by subcloning a gene-synthesized *BsrGI-NotI* fragment (Genewiz) in which the shRNA recognition sequence was mutated while maintaining the protein coding sequence. CCSS mutation was introduced by Quickchange mutagenesis. For studies in DRG neurons, the EF1 alpha promoter was replaced with the human synapsin (hSyn) promoter, generating a vector termed FSW (49), to ensure neuron-specific JAK1 expression. Human STAT3 cDNA was also obtained from DNASU and was subcloned into myc-FEW and pCIS-GST vectors, the latter vector allowing the production of GST-tagged STAT3 fusion protein in mammalian cells. Mouse *Zdhhc* cDNAs were a kind gift of Dr Masaki Fukata and were used in HA-FEW vector as described (7). ShRNA sequences against rat *Zdhhc3* (5′- GAGACATTGAACGGAAACCAGAATACCTC-3′) and *Zdhhc7* (5′- ATGACATGGCTTCTGGTCGTCTATGCAGA-3′) were purchased from Origene and subcloned, together with their neighboring U6 cassette, into FEGW. These shRNAs are designated as *Zdhhc3* and *Zdhhc7* sh1. A second pair of *Zdhhc3* (5′-AAGAGAGAAGATGGGCTATT-3′) and *Zdhhc7* (5′-AGGAGTACATGGAGAGCTTTT-3′) shRNAs were identified bioinformatically and cloned into the same FEGW vector, but with a neighboring H1 promoter. These shRNAs are designated as *Zdhhc3* and *Zdhhc7* sh2. VSV-G pseudotyped lentiviruses were prepared in HEK293T cells as described (13).

### Acyl biotin exchange assay

Palmitoylation was detected by ABE assay as described (13). Briefly, HEK293T cells or DRG neurons were lysed in ABE lysis buffer (50 mM HEPES, pH 7, 2% [w/v] sodium dodecyl sulfate (SDS), 1 mM EDTA plus protease inhibitors (PIC)), and 20 mM thiol-reactive methyl-methane thiosulfonate (MMTS), sonicated, and incubated at 50 °C for 20 min. Protein was precipitated by the addition of chilled acetone (80% final [v/v]), and MMTS was removed by sequential washes with 80% [v/v] acetone. Pellets were resuspended in 4% SDS buffer (4% [w/v] SDS, 50 mM Tris pH 7.5, 5 mM EDTA plus PIC) and a fraction was removed as an “Input” sample. Inputs were taken out in dilution buffer (50 mM HEPES, 1% [v/v] Triton X-100, 1 mM EDTA, 1 mM EGTA) plus PIC, 150 mM NaCl, and 5x sample buffer with β-mercaptoethanol (BME) (Millipore-Sigma). Samples were split in two and incubated for 1 h rotating in the dark at room temperature in 1x Protease Inhibitor Cocktail (Boehringer), 1 mM HPDP-biotin (Soltec Ventures, Beverly, MA), 0.2% Triton X-100, with either 1M hydroxylamine pH 7.5, or 50 mM Tris pH 7.5. Samples were acetone-precipitated to remove hydroxylamine/Tris and HPDP-biotin and pellets were resuspended in ABE lysis buffer plus PIC and diluted 1:20 in dilution buffer plus 150 mM NaCl and protease inhibitors. Biotinylated proteins were captured by incubation for 3 h with high capacity neutravidin-conjugated beads (Thermo Fisher Scientific) at 4 °C. Beads were washed three times with dilution buffer containing 0.5 M NaCl, and twice with dilution buffer alone. Protease inhibitors (4 μg/ml Leupeptin and 1 mM Benzamidine) were added to all washes. Proteins were eluted from beads by addition of 1% (v/v) BME, 0.2% [w/v] SDS, 250 mM NaCl in dilution buffer and incubated for 10 min at 37 °C. Supernatants were removed and denatured by adding one-fifth volume of 5× SDS sample buffer. Samples were boiled and subjected to SDS-PAGE and Western blotting.

### RNA extraction and quantitative RT-PCR

Total RNA was isolated from cultured embryonic DRG neurons using the RNeasy micro kit (Qiagen). 200 ng of total RNA was used to prepare cDNA using the SuperScript III First-Strand Synthesis System (Life Technologies). The cDNA was diluted 1:5 and quantitative PCR (qPCR) was performed on the rat *Zdhhc3* and *Zdhhc7* genes using primers listed below and the Power SYBR Green PCR Master Mix (Applied Biosystems) in a StepOnePlus Real-Time PCR System under default conditions. Each sample was run in triplicate. Expression levels for mRNA were normalized to a normalization factor of *Actnb*, *Rpl13a*, and *Hprt* expression in the same samples. Primers used were as follows: *Zdhhc3* exons 5/6 spanning forward ACCCAAGTGCACTCCATCTGC and reverse CCAGCCTAAAGAGAAAGGGTGG [this study]; *Zdhhc7* exons 4/5 spanning forward GCTTCTTCGTGCTCTTCACC and reverse AGGCACAGGAAGACCAACAG [this study]; *Actnb* exons 2/3 spanning forward CCGCGAGTACAACCTTCTTG and reverse CGCAGCGATATCGTCATCCAT [this study]; *Hprt* exons 5/6 spanning forward ACCAGTCAACGGGGGACATA and reverse TTGGGGCTGTACTGCTTGAC [this study]; *Rpl13a* forward GGATCCCTCCACCCTATGACA and reverse CTGGTACTTCCACCCGACCTC (50).

### GST-STAT3 purification

HEK293T cells were transfected with pCIS-GST-STAT3 vector and cells were lysed 24 h later in immunoprecipitation buffer (IPB: 1x phosphate-buffered saline [PBS] pH7.4, 1% [w/v] Triton X-100, 50 mM NaF, 5 mM Na_4_P_2_O_7_, 1 mM Na_3_VO_4_, 1 mM EDTA, and 1 mM EGTA) plus 1x PIC. Lysates were centrifuged at 13,000*g* and supernatants were incubated with Glutathione Sepharose beads (pre-equilibrated with IPB) for 90 min at 4 °C on a rotating platform. Beads were then washed four times with IPB plus 0.25 M NaCl, twice with IPB, and once with 50 mM Tris pH 7.5. All wash buffers contained protease inhibitors (4 μg/ml Leupeptin and 1 mM Benzamidine). GST-tagged STAT3 was eluted by the addition of 20 mM Glutathione pH 8.0 plus 250 mM NaCl, was dialyzed extensively against 50 mM Tris pH 7.5 containing 50%(v/v) glycerol and was stored unfrozen at −20 °C. The preparation (judged to be >80% pure by Coomassie staining) was used for subsequent *in vitro* kinase assays.

### Immunoprecipitation-kinase activity assay

HEK293T cells transfected to express HA-tagged JAK1WT or -CCSS were treated with or without 20 μM 2Br and lysed in IPB. Lysates were spun at 13,000 rpm for 10 min at 4 °C. Supernatant was then filtered through a 0.22 μm SpinX column. A fraction of each sample was denatured in an SDS sample buffer for use as “Inputs”. 200 μL of the remaining lysate was incubated on a rotating platform for 90 min at 4 °C with 5 μl of Protein G Sepharose beads (settled volume) that had been pre-coupled with 1 μl of anti-HA ascites. Beads were washed twice with IPB containing 0.5 M NaCl, twice with IPB alone and once with 1× Kinase assay buffer (10 mM Tris pH 7.5, 0.2 mM EDTA, and 0.1% TritonX-100). Excess supernatant was removed and beads were equilibrated in 20 μL (total volume) of 1× Kinase Assay Buffer containing 1 μM GST-STAT3. Reactions were started by the addition of 5 μl of 0.5 mM ATP, 50 mM MgCl_2,_ and a fraction of the reaction mix was immediately removed as a t = 0 sample and stopped by dilution in 1× SDS sample buffer. Reactions were then allowed to proceed at room temperature with regular flick-mixing. Fractions of the reaction were removed at defined times and stopped by dilution in 1× SDS sample buffer, boiled for 5 min and subjected to SDS-PAGE and western blotting.

### Structural modeling

The protein sequence of human JAK1 was taken from Uniprot (accession P23458). Modeling with AlphaFold2-ptm (51) and AlphaFold-Multimer (29) was performed with the Colabfold Jupyter notebook (52). Each AlphaFold program is in reality a set of five different sets of model parameters (weights in the deep learning neural network model). Four random seeds were used for modeling the monomer and homodimer of JAK1, resulting in 20 monomeric models and 20 dimeric models. Templates from the Protein Data Bank were not used. All other parameters were the default values.

### Immunocytochemistry

Dissociated DRG neurons cultured on coverslips were rinsed once with 1x Recording buffer (25 mM HEPES pH7.4, 120 mM NaCl, 5 mM KCl, 2 mM CaCl_2_, 1 mM MgCl_2_, 30 mM Glucose) and fixed in 4% paraformaldehyde (PFA)/sucrose for 10 min at room temperature. Samples were permeabilized in PBS containing 0.25% (w/v) Triton-X-100 for 10 min at 4 °C, blocked with PBS containing 10% (v/v) Normal Goat Serum (Southern Biotech, 0060-01) for 1 h and incubated overnight at 4 °C with primary antibodies diluted in blocking solution. After three washes with PBS, cells were incubated for 1 h at room temperature with AlexaDye-conjugated fluorescent secondary antibodies diluted in blocking solution, prior to three final PBS washes and mounting in FluorSave reagent (Millipore Sigma).

### Image acquisition

Images were acquired using a Nikon C2 confocal microscope (60×, 1.4NA oil objective for images in Fig. 2; 10×, 0.3NA objective for images in Fig. 7) and NIS Elements software. Files were exported and quantified using Fiji/ImageJ. Images shown are maximum intensity projections of confocal Z-stacks. Images were not taken by an experimenter blinded to treatment condition. For images in Figure 2, fields of view were selected based only on GFP signal (marker of infected neurons) without reference to the HA signal (JAK1 “rescue” constructs) prior to acquisition. For images in Figure 7, the objective was moved to the “12 o’clock” position of each coverslip and moved directly “down” the coverslip towards the center, with images acquired from directly adjacent but non-overlapping fields. This procedure was followed for all conditions and for all technical and biological replicates.

### Experimental replicates

All experiments with cultured neurons were repeated from at least three separate dissections. For most neuronal biochemical experiments, each data point plotted represents the result from an individual well from a separate dissection (*i.e.* a single biological replicate or “n”). In some instances, duplicate wells from a single dissection (technical replicates) were assessed in parallel and the result was averaged to give a single biological replicate. For ABE assays from neurons, equal numbers of individual wells per condition were pooled and used to generate a single biological replicate, which is then plotted. For neuronal immunocytochemical experiments, 3 to 4 images were acquired per coverslip and quantified and the result was averaged to generate a single biological replicate (“n”), which is then plotted. For experiments in HEK293T cells, each data point represents a single plate or well. Where data from HEK293T cells are not quantified, the experiment was performed at least three times, from at least two separate transfections, and representative results are shown.

## Data availability

All primary data that are not directly presented can be obtained from the lead contact (gareth.thomas@temple.edu) upon reasonable request.

## Supporting information

This article contains supporting information.

## Conflict of interest

The authors declare that they have no conflicts of interest with the contents of this article.
