# Supplementary material for: Palmitoylation-dependent control of JAK1 kinase signaling governs responses to neuropoietic cytokines and survival in DRG neurons
Source: J Biol Chem. 2023 Jun 24;299(8):104965. doi: 10.1016/j.jbc.2023.104965 (PMC10413081; doi:10.1016/j.jbc.2023.104965)
Supplement: Supporting Figures S1–S3 [file mmc1.docx]

**Supporting information for:**

**Palmitoylation-dependent control of JAK1 kinase signaling governs responses to neuropoietic cytokines and survival in DRG neurons**

by

Luiselys M. Hernandez, Audrey Montersino, Jingwen Niu, Shuchi Guo, Bulat Faezov, Shaun S. Sanders, Roland L. Dunbrack and Gareth M. Thomas

**
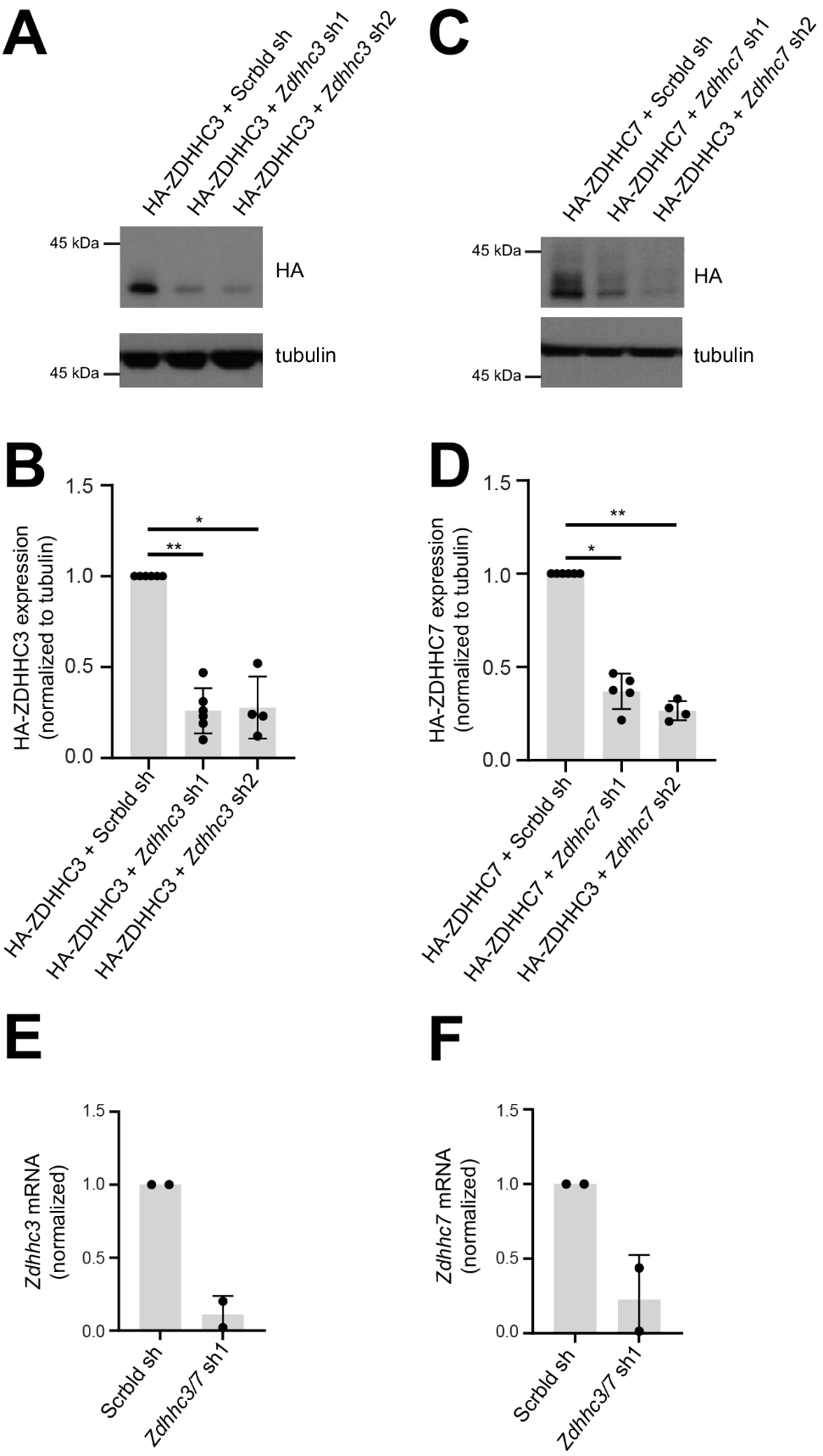
**

**Figure S1:** **Confirmation of Efficacy of *Zdhhc3* and *Zdhhc7* shRNAs. *A:*** Western blots of lysates from HEK293T cells that had been cotransfected to express HA-tagged rat ZDHHC3 plus either a scrambled (Scrbld) shRNA or the indicated *Zdhhc3* shRNA. ***B:*** Quantified data from *A.* Both *Zdhhc3* shRNAs significantly reduce HA-ZDHHC3 expression. **;p<0.01, *;p<0.05, non-parametric one-way ANOVA with Dunn’s multiple comparison test, Kruskal-Wallis statistic 11.18, p=0.0006. ***C:*** Western blots of lysates from HEK293T cells that had been cotransfected to express HA-tagged rat ZDHHC7 plus either Scrbld shRNA or the indicated *Zdhhc7* shRNA. ***D:*** Quantified data from *C.* Both *Zdhhc7* shRNAs significantly reduce HA-ZDHHC7 expression. **;p<0.01, *;p<0.05, non-parametric one-way ANOVA with Dunn’s multiple comparison test, Kruskal-Wallis statistic 11.98, p<0.0001. ***E:*** *Zdhhc3* mRNA levels in DRG neurons lentivirally infected to express the indicated shRNAs, normalized to a normalization factor of *Actnb*, *Rpl13a*, and *Hprt* mRNA expression and plotted relative to the Scrbld shRNA condition. Data are from 2 biological replicates per condition. ***F:*** As *E*, but for *Zdhhc7* mRNA levels.

**
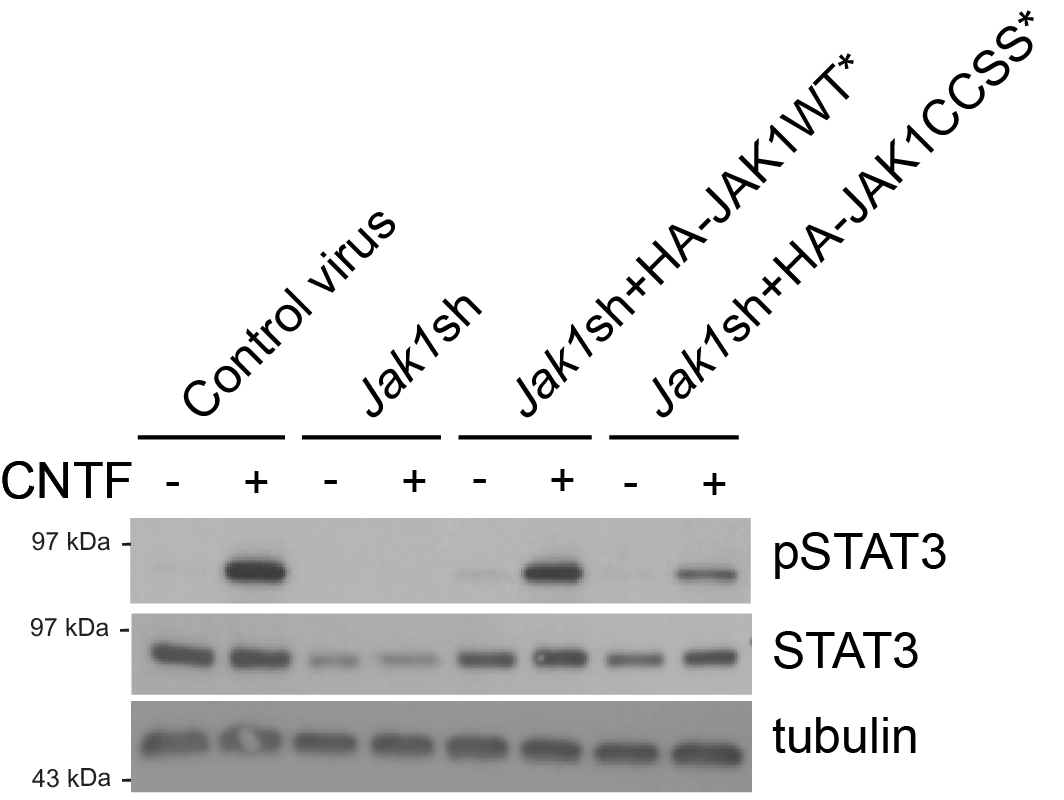
**

**Figure S2: Palmitoylation of JAK1 is important for CNTF-dependent STAT3 phosphorylation in cultured DRG neurons.** Western blots of lysates of DRG neurons that had been cultured in the continued presence of NGF to ensure survival, infected with the indicated lentiviruses and subsequently stimulated with CNTF. Representative of three independent experiments. Similar expression of shRNA-resistant JAK1WT and JAK1CCSS was confirmed as in Fig 2A.

**
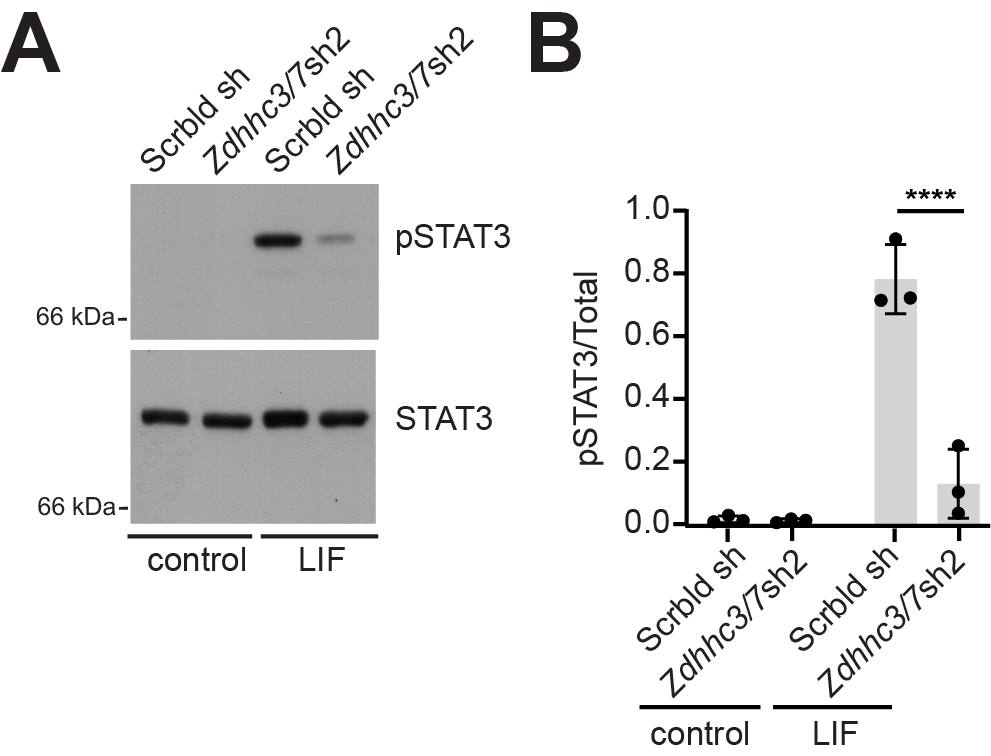
**

**Figure S3:** **A second pair of *Zdhhc3/Zdhhc7* shRNAs reduces LIF-induced STAT3 phosphorylation in DRG neurons. *A:*** Western blots from DRG neurons lentivirally infected to express scrambled sh (Scrbld sh) or *Zdhhc3/7* sh2, stimulated with LIF or left unstimulated and immunoblotted with the indicated antibodies (see also Fig S1). ***B:*** Quantified data from *A* confirm reduced LIF-induced STAT3 phosphorylation in *Zdhhc3/*7 knockdown neurons. ****: p<0.0001, two-way ANOVA with Tukey test. N = 3 per condition. Virus condition p<0.0001 (F(1, 8) = 53.02), treatment p<0.0001 (F (1, 8) = 95.72), interaction p<0.0001 (F(1, 8) = 51.52).
